# Insulin Resistance Promotes the Formation of Aortic Dissection by Inducing the Phenotypic Switch of Vascular Smooth Muscle Cells

**DOI:** 10.3389/fcvm.2021.732122

**Published:** 2022-02-03

**Authors:** Hui Zheng, Zhihuang Qiu, Tianci Chai, Jian He, Yuling Zhang, Chaoyun Wang, Jianqiang Ye, Xiaohui Wu, Yumei Li, Li Zhang, Liangwan Chen

**Affiliations:** ^1^Department of Cardiovascular Surgery, Fujian Medical University Union Hospital, Fuzhou, China; ^2^Key Laboratory of Cardio-Thoracic Surgery (Fujian Medical University), Fuzhou, China; ^3^Fujian Provincial Special Reserve Talents Laboratory, Fujian University, Fuzhou, China; ^4^Engineering Research Center of Tissue and Organ Regeneration, Fujian University, Fuzhou, China; ^5^Fujian Center for Evaluation of New Drug, Fujian Medical University, Fuzhou, China; ^6^Department of Physiology and Pathophysiology, School of Basic Medical Sciences, Fujian Medical University, Fuzhou, China

**Keywords:** insulin resistance, phenotypic switch, aortic dissection, vascular smooth muscle cells, promotes

## Abstract

**Background:**

Insulin resistance (IR) plays a key role in the development of type 2 diabetes mellitus (T2DM) and is one of its most important characteristics. Previous studies have shown that IR and T2DM were independent risk factors for a variety of cardiovascular and cerebrovascular diseases. However, there are few studies on the relationship between IR and aortic dissection (AD). The goal of this research was to find evidence that IR promotes the occurrence of AD.

**Methods:**

Through the statistical analysis, we determined the proportion of glycosylated hemoglobin (HbA1c) abnormalities (HbA1c > 5.7) in people with acute thoracic aortic dissection (ATAD) and compared the difference of messenger RNA (mRNA) and protein expression of GluT1 in the thoracic aorta of normal people and those with ATAD to find evidence that IR is a causative factor in AD. The mouse model of IR and AD and the IR model of human aortic vascular smooth muscle cells (HA-VSMC) were established. Real time-PCR (RT-PCR) and Western blotting were used to study the mRNA and protein expression. Hematoxylin and eosin (H&E), Masson, and elastic fiber staining, and immunofluorescence were used to study the morphological structure.

**Results:**

The proportion of HbA1c abnormalities in patients with ATAD was 59.37%, and the mRNA and protein expression of GluT1 were significantly lower than that in normal people. Fasting glucose concentration (FGC), serum insulin concentration (SIC), and the homeostasis model assessment of insulin resistance (HOMA-IR) of mice was obviously increased in the high-fat diet group and the protein expressions of Glut1 and GluT4 were reduced, indicating that the mouse IR model was successfully established. The incidence of AD was different between the two groups (IR: 13/14, Ctrl: 6/14), and the protein expression of MMP2, MMP9, and OPN were upregulated and SM22 and α-SMA were downregulated in mice. The expressions of mRNA and protein of GluT1 and SM22 in HA-VSMCs with IR were reduced and OPN was increased.

**Conclusion:**

Combined results of clinical findings, mouse models, and cell experiments show that IR induced the phenotypic switching of vascular smooth muscle cells (VSMCs) from contractile to synthetic, which contributes to the occurrence of AD. It provides a basis for further research on the specific mechanism of how IR results in AD and a new approach for the prevention and treatment of AD.

## Introduction

Acute aortic dissection (AAD) is a life-threatening disease, and without treatment, the fatality rate rises by 1–2% per hour after the onset of symptoms (1, 2). With the development of diagnostic imaging technology, it can be identified and typed quickly and accurately. Surgical repair is the first choice for type A aortic dissection (AD), and type B is treated mainly by drugs or stent endovascular repair (3). However, the mortality in severe complications caused by vascular tear damage is still very high (4).

Previous studies showed that gender (5), age (4), hypertension (2, 6), aneurysm (7, 8), arteritis (9, 10), and atherosclerosis (11) were independent high-risk factors for acute thoracic aortic dissection (ATAD) and promote its occurrence and development. However, most clinical studies have shown that diabetes was negatively correlated with the occurrence of AD (12–15) and was also inversely related to the rupture of aneurysm and enlargement of the aneurysm volume (16–18).

Insulin resistance (IR) accompanies the occurrence and development of type 2 diabetes mellitus (T2DM), which is one of its most important features and is related to atherosclerotic diseases such as coronary artery disease, cerebrovascular disease, carotid artery stenosis, and peripheral vascular disease (19). Studies have shown that C-peptide, serum insulin concentration (SIC), and the homeostasis model assessment of insulin resistance (HOMA-IR) were significantly upregulated in patients with a larger aneurysm diameter (20), and HbA1c was a high-risk factor for AD (21). There have been few studies on the relationship between IR and AD. IR affects nutrient metabolism, inflammatory infiltration, and the release and activation of inflammatory cytokines, which is why we consider that IR is a causative factor of AD.

Through epidemiological investigations, we found that IR may be a high-risk factor for ATAD, and the discrepancies of the messenger RNA (mRNA) and protein expression of GluT1 in thoracic aortic smooth muscle cells (TASMC) between normal and patients with ATAD confirmed this. The IR models of mouse and human aortic vascular smooth muscle cells (HA-VSMCs) indicate that IR induced the phenotypic switching of vascular smooth muscle cells (VSMCs) from contractile to synthetic, which promotes the occurrence of AD. This provides evidence for further research on the definite mechanism and a new approach for the prevention and treatment of AD.

## Materials and Methods

### Cell Culture and Establishment of the HA-VSMC IR Model

The HA-VSMC line T/G HA-VSMC (HTX2061) was maintained and cultured in Dulbecco's Modified Eagle's medium (DMEM) supplemented with 10% fetal bovine serum (FBS) (PAN Biotech, Germany) and 1% penicillin/G-streptomycin sulfate in a 5% CO_2_ humidified atmosphere with a constant temperature of 37°C. The T/G HA-VSMCs IR model was established by culturing cells in DMEM containing 10% FBS (10^−5^, 10^−6^, 10^−7^, 10^−8^ mol/L), insulin, and 1% penicillin/G-streptomycin sulfate after 24 h of serum depletion in a 5% carbon dioxide (CO_2_) humidified atmosphere with a constant temperature of 37°C. The glucose oxidase method (Glucose Determination Kit, Rubio, China) was used to detect the consumption of glucose in the culture medium, and the number of cells was detected by the Cell-Counting Kit-8 (Do Jindo, Japan). The glucose consumption of the same cells was used to screen out the insulin concentration and the action time of the highest state of IR.

### Development of the Mouse Model

Three-week-old AopE^−/−^ male mice (C57BL/6 background) were purchased from GenPharmatech Co. Ltd. (Nanjing, China). All mice were randomly divided into two groups and fed a regular diet [control group: normal diet; IR group: 60% high-fat diet (D12492)] and given beta-aminoproprionitrile (BAPN) (Sigma-Aldrich, St. Louis, MO, USA) dissolved in drinking water (0.25%) for 4 weeks. At 7 weeks of age, osmotic mini-pumps (Alzet, Cupertino, CA, USA) delivering 1 ug/kg/min Ang-II (Sigma-Aldrich) were implanted subcutaneously, and the mice were euthanized 24 h after implantation. Fresh aortic tissue was obtained, liquid nitrogen was used to maintain the samples for protein verification, and 4% paraformaldehyde was used to fix specimens before paraffin embedding.

Fasting glucose concentration (FGC) was measured using reagent strips read in a glucose meter (YSI 2300-STAT), and SIC was measured using mouse insulin (INS) ELISA-kits (CSB-E05071m, CUSABIO, China) by the tail-cuff method after grouping and before implantation. The the homeostasis model assessment of insulin resistance (HOMA-IR) was calculated to evaluate insulin resistance (HOMA-IR=[${\text{FGC}}_{*}\text{SIC}/22.5$]).

All studies in mice were approved by the Laboratory Animal Care and Use Committee of the School of Fujian Medical University (No. 2020-0089).

### Epidemiological Investigation

We collected data on patients with ATAD in cardiac surgery from July 2017 to June 2020 in Fujian Medical University Union Hospital and excluded patients with Marfan syndrome, aortic arteritis, type 1 diabetes a history of syphilis, and excluded those who had not undergone HbA1c testing at the time of admission. In total, 3,185 participants were identified, and a total of 552 patients met the requirements.

### Human Thoracic Aorta Tissues Samples

Normal thoracic aorta tissue from heart transplant donors was discarded during the operation. The thoracic aorta tissues of the ATAD groups were obtained from the tissues removed during thoracic aortic replacement surgery.

Samples of the intima and externa were removed and washed with cold phosphate buffer solution (PBS), then placed in liquid nitrogen, an RNA protection solution, and 4% paraformaldehyde, respectively, for subsequent protein and RNA extraction and paraffin embedding.

All procedures were approved by the Ethics Committee at the Fujian Medical University Union Hospital (No. 2021QH026).

### Western Blot Analysis

Proteins were extracted from human aortic tissue, mouse aortic tissue, and HA-VSMCs using a radioimmunoprecipitation assay (RIPA) buffer containing phenylmethylsulfonyl fluoride (PMSF) (Bio Sharp, China). The total protein levels of each sample were quantified using the bicinchoninic (BCA) protein assay (Beyotime Biotechnology, China). Proteins were separated on SDS-PAGE gel electrophoresis and transferred to polyvinylidene fluoride (PVDF) (Bio Sharp) membranes. Membranes were placed in 5% skimmed milk for 1 h at room temperature and then incubated with various primary antibodies (Table 1) overnight at 4°C. Subsequently, secondary antibody incubations were carried out at 37°C for 1 h. Signals were detected and quantified using the Chemiluminescent HRP Substrate (Millipore, USA) and the band density was quantified with Image J (NIH, USA).

**Table 1:** Antibodies used.

**Antibody**	**Manufacturer**	**Catalog number**	**Source**	**Dilutions**
GluT1	Immunoway	YT1928	Rabbit	1:1,000
GluT4	Immunoway	YT5523	Rabbit	1:1,000
SM22	Proteintech	60213-1-lg	Mouse	1:5,000
α-SMA	Bioss	Bs-10196R	Rabbit	1:10,000
OPN	BOSTER	BM4208	Rabbit	1:1,000
MMP2	Proteintech	66366-1-lg	Mouse	1:5,000
MMP9(anti-human)	Abcam	ab76003	Rabbit	1:2,500
MMP9(anti-mouse)	Abcam	ab228402	Rabbit	1:1,000
β-actin	BOSTER	BM0627	Mouse	1:10,000
Goat anti-mouse	Immunoway	RS0001	Goat	1:10,000
Goat anti-rabbit	Immunoway	RS0002	Goat	1:10,000

### RT-qPCR

Real-time- qPCR (RT-qPCR) was used to detect the gene expression levels of GluT1, GluT4, MMP-9, MMP-2, OPN, SM22, and α-SMA (Table 2). RNA was isolated using the TRIzol Reagent (Ambion, USA) and reverse transcribed using the reverse transcription kit (Takara, Japan) to determine these gene expression levels. β-actin was applied as an internal control for RNA-related experiments. The PCR primers are shown in Table 1. RT-qPCR was performed using SYBR green dye on the Biosystems 7500 Real-Time PCR System (Applied Biosystems, USA). Manufacturer protocols provided by Prime Script^TM^ RT-PCR Kit and the Fast Start Essential DNA Green Master (Roche, Germany) were used.

**Table 2:** Primer sets.

**Gene**	**Forward primer**	**Reverse primer**
MMP2	5′-CTGTTGCTGCCCATCTGAAG-3′	5′-AGAATGATGGGCACTACCGTG-3′
MMP9	5′-TTGACAGCGACAAGAAGTGG-3′	5′-CCTCAGTGAAGCGGTACATAG-3′
OPN	5′-TGAGCATTCCGATGTGATTGAT-3′	5′-GGTCTACAACCAGCATATCTTCAT-3′
SM22	5′-AGAATGATGGGCACTACCGTG-3′	5′-CTGTTGCTGCCCATCTGAAG-3′
GluT1	5′-TCTGGCATCAACGCTGTCTT-3′	5′-CCGTGTTGACGATACCGGAG-3′
β-actin	5′-TGACGTGGACATCCGCAAAG-3′	5′-CTGGAAGGTGGACAGCGAGG-3′

### Immunofluorescence

Human and mouse thoracic aorta tissues were embedded with paraffin and sliced into 2.5-μm thick sections. Paraffin slides were treated in an oven (65°C) and soaked in xylene and alcohol with different concentration gradients for dewaxing, followed by 3% H_2_O_2_ for 20 min and blocked with goat serum. Further, the slides were immune-stained with antibodies against GluT1 (Immunoway, Catalog NO:YT1928, 1:100) overnight at 4°C. Subsequently, secondary antibody (Alexa Fluor555-labeled donkey anti-rabbit IgG, Beyotime, Catalog NO: A0453, 1:500) incubations were carried out at 37°C for 1 h. 4',6-Diamidino-2-phenylindole dihydrochloride (DAPI) fluorescent stain was used to stain the nucleus for 15 min. These sections were mounted and visualized under a Nikon fluorescence microscope (Nikon ECLIPSE Ni).

The HA-VSMCs were seeded in six-well Nunc Lab-Tek Chamber Slide (Thermofisher, Catalog #177399) and cells were treated with DEMM and 10^−7^ mol/L insulin after 24 h of serum depletion. After treatment for 36 h, cells were washed with phosphate-buffered saline (PBS) and fixed with 4% PFA, and permeabilized with 0.2% Triton-X 100 in 1X PBS for 15 min. Further, cells were immune-stained with primary antibodies (GluT1, Immunoway, Catalog YT1928, 1:100; SM22, Proteintech, Catalog 60213-1-Ig, 1:100; and OPN, BOSTER, Catalog BM4208, 1:50) overnight at 4°C and secondary antibodies (Alexa Fluor555-labeled donkey anti-rabbit IgG, Beyotime, Catalog No.: A0453, 1:500 and FITC-labeled goat anti-mouse IgG, Beyotime, Catalog No.:A0568, 1:500) incubations were carried out at 37°C for 1 h. DAPI was used to stain the nucleus for 15 min and samples were mounted and visualized by a Nikon fluorescence microscope (Nikon ECLIPSE Ni).

### Histopathological Analysis

Complete gross and histopathological evaluations were performed with samples from the control and IR group mice. After euthanasia, aortic tissues were harvested from the thoracic aorta and were fixed in 4% paraformaldehyde, then were sectioned at 2.5-μm thickness after fixation and paraffin embedding, stained with hematoxylin and eosin (H&E) following standard procedures, and examined under light microscopy.

### Elastin Staining

Elastin in the normal and dissected aortas was stained with Verhöeff elastic fiber dyeing liquid using an elastic fiber staining kit (Yuanye Bio-Technology Co. Ltd., Shanghai, China), according to the manufacturer's instructions.

### Masson Staining

The collagenous fiber was stained with Masson's trichrome stain kit (Beijing Solar Bioscience and Technology Co. Ltd, Beijing, China), according to the manufacturer's instructions.

### Statistical Analysis

All data are expressed as mean ± SEM. Unpaired *t*-tests were used to analyze the differences between the two groups, one-way ANOVA was applied to assess the differences among comparisons of more than two groups. Prism 8.0 software (GraphPad, San Diego, CA, USA) was used for statistical analysis. All data were analyzed using two-tailed tests and *P* < 0.05 were defined as statistically significant: *P* < 0.05 ^*^; *P* < 0.01 ^**^; *P* < 0.001 ^***^; *P* < 0.0001 ^****^.

## Results

### Clinical Phenomenon and Human Thoracic Aortic Tissue Verification Results

**Figure 1:**
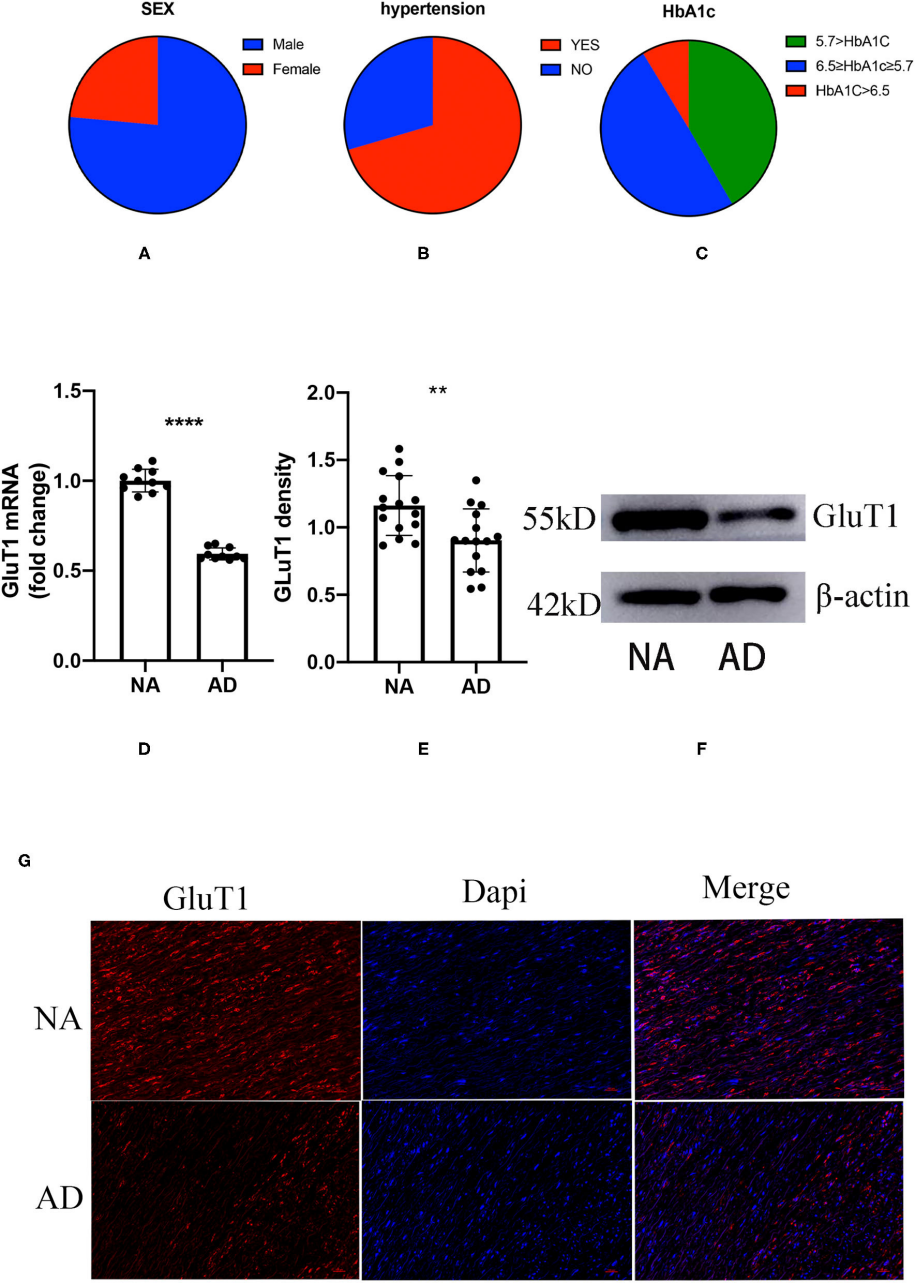
The proportion of gender, hypertension, and glycosylated hemoglobin (HbA1c) in patients with acute thoracic aortic dissection (ATAD); the difference between aortic dissection (AD) and donors (NA) in the GluT1 expression in TAVSMCs. **(A)** M/F:76.45%:/23.55%; **(B)** hypertension/no-hypertension: 70.47%/29.53%; **(C)** HbA1c ≥ 6.5, 8.7%; 6.5 > HbA1c ≥ 5.7, 49.64%; HbA1c < 5.7, 41.67%; **(D)** qPCR analysis of GluT1mRNA levels, ****P < 0.0001. **(E,F)** WB analysis of GluT1 protein levels, **P < 0.01. **(G)** Immunofluorescence staining of GluT1 (red) in aortic human vascular smooth muscle cell (HVSMC) cells which from AD and NA, DAPI staining was used to visualize cell nuclei. Scale bar = 100 μm.

The homeostasis model assessment of insulin resistance (HOMA-IR) is the most common index used for clinical evaluation of insulin resistance, but the patients with ATAD require emergency surgery, and it is difficult to collect FGC and SIC. However, HbA1c is not affected by diet, so we choose HbA1c as an indirect indicator of IR to reflect the extent of IR in patients with ATAD. Of our subjects, 76.45% were male, age 54.12 ± 12.13 years, and 23.55% were female, age 56.77 ± 11.18 years among the patients who met the criteria (Figure 1A). There were 70.47% with a history of hypertension (Figure 1B); HbA1c ≥ 6.5 8.7%, HbA1c < 5.7:41.67%, 6.5 > HbA1c ≥ 5.7:49.64% (Figure 1C). According to the diagnostic criteria for IR, we found that the majority of patients with AD simultaneously had IR.

To clarify the relationship between ATAD and IR, we used quantitative PCR (qPCR) to detect the expression of GluT1 in ascending aortic vascular smooth muscle. The results showed that the mRNA expression of GluT1 in VSMC of patients with ATAD was significantly reduced (*P* < 0.0001, Figure 1D)

GluT1 is a functional membrane protein that transports glucose into cells for metabolism. Using western blot technology, we found that the GluT1 of the TASMCs of patients with ATAD was significantly lower than that of normal people (*P* < 0.01) (Figures 1E,F). Moreover, we obtained a similar result using immunofluorescence that showed that GluT1 is reduced (Figure 1G). Based on these experimental results, we believe that IR is a high-risk factor for AAD.

### Animal Studies

#### A High-Fat Diet Can Increase FGC, SIC, and HOMA-IR in Mice

**Figure 2:**
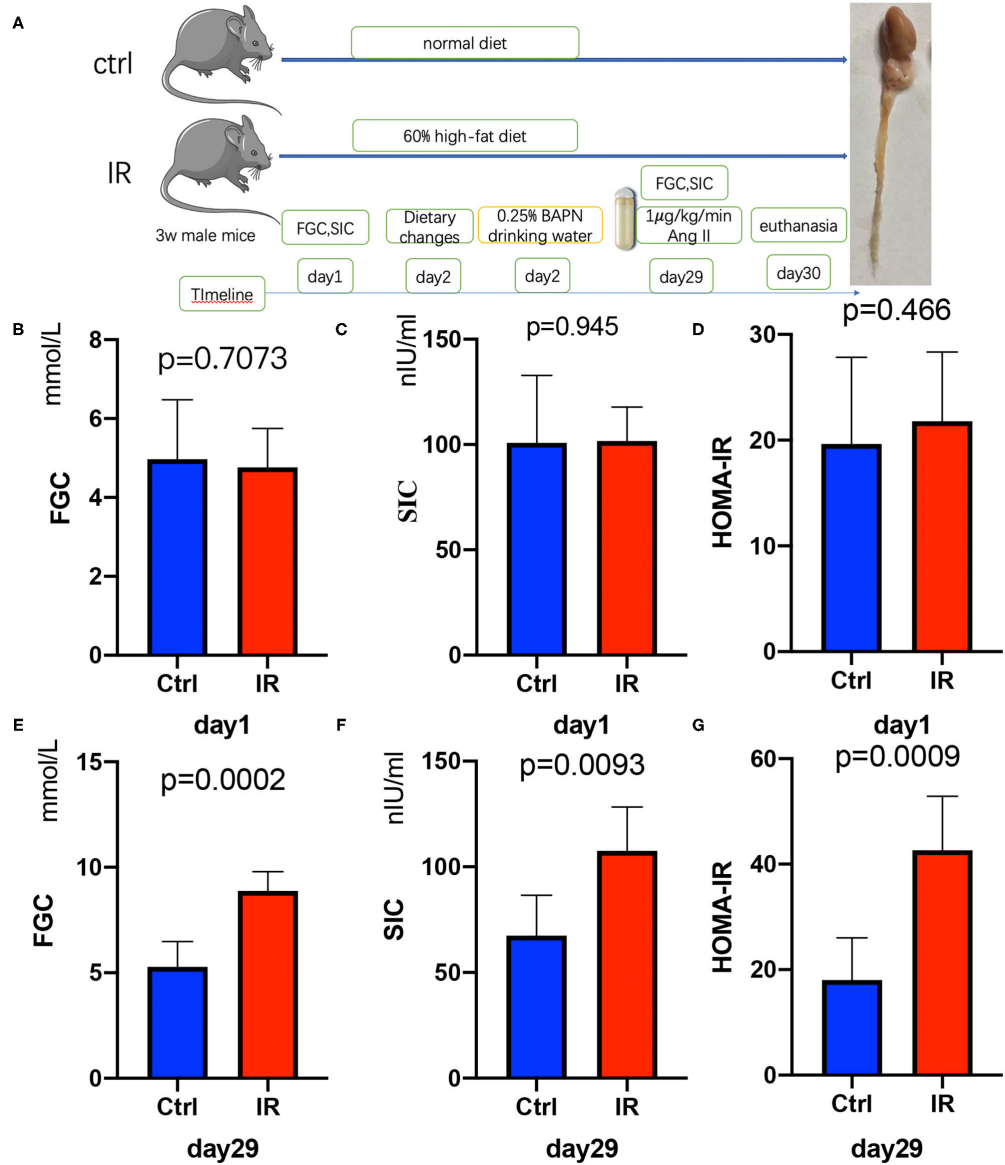
Establishment of insulin resistance (IR) and AD model in ApoE^−/−^ mice, Test fasting glucose concentration (FGC), serum insulin concentration (SIC), and calculate homeostasis model assessment of insulin resistance (HOMA-IR). **(A)** The flow diagram of animal experiments. **(B–D)** The FGC (*P* =.7073), SIC (*P* = 0.945), and HOMA-IR (*P* = 0.466) on day 1 in the two groups. **(E–G)** The FGC (*P* = 0.0002), SIC (*P* = 0.0093), and HOMA-IR (*P* = 0.0009) on day 29.

After the ApoE^−/−^ mice were quarantined and adapted, they were randomly divided into two groups (Figure 2A). The FGC was measured after 12 h of starvation and the tail vein blood was collected to obtain SIC. The HOMA-IR was calculated. There was no difference between the two groups in FGC, SIC, and HOMA-IR (*P* = 0.7073, *P* = 0.945, *P* = 0.466) (Figures 2B–D). After 4 weeks of feeding with different diets, it was found that FGC, SIC, and HOMA-IR in the high-fat diet group were significantly increased, all higher than in the ordinary diet group (*P* = 0.0002, *P* = 0.0093, *P* = 0.0009) (Figures 2E–G).

#### A High-Fat Diet Induces Decreased Expression of GluT1 and GluT4 Decline in the VSMCs in ApoE^–/–^ Mice

**Figure 3:**
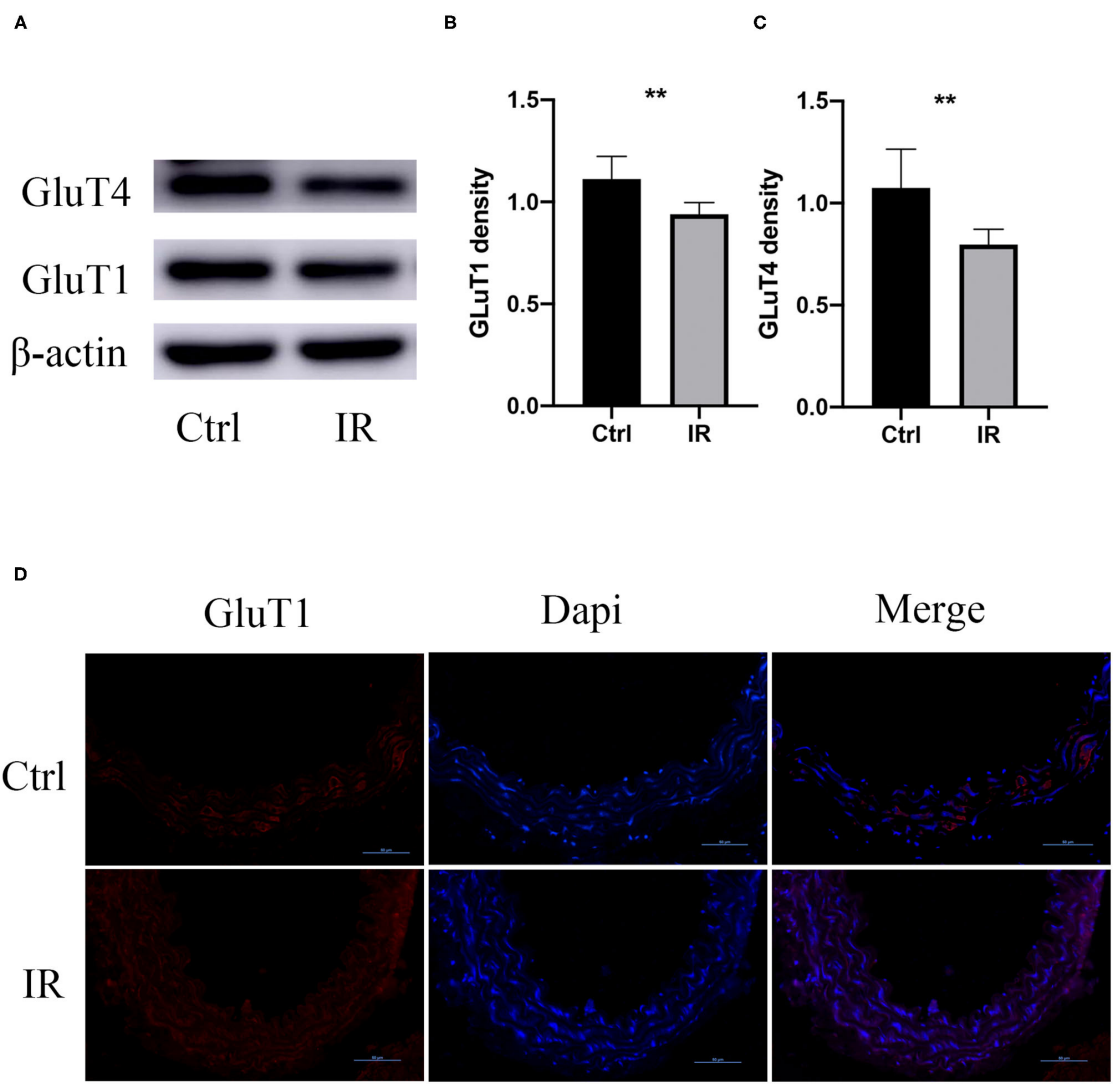
GluT1 and GluT4 protein expression in mouse aortic tissue. **(A–C)** WB analysis of GluT1 and GluT4 protein levels, **P < 0.01, **P < 0.01. **(D)** Immunofluorescence staining of GluT1 (red) in aortic MSMC cells from IR and control groups. DAPI staining was used to visualize cell nuclei. Scale bar = 50 μm.

To determine whether there was IR in mouse aortic tissue, we collected fresh aortic tissue of the mice, extracted the proteins, and used western blot to analyze the protein expression of Glut1 and GluT4 in the two groups. The results showed that the protein expression of Glut1 and GluT4 in the intervention group was significantly lower than that of the normal diet group (*P* < 0.01, *P* < 0.01) (Figures 3A–C), and results were further confirmed by immunofluorescence (Figure 3D).

#### The Incidence of AD in the IR Group Mice Was Higher Than in the Control Group

**Figure 4:**
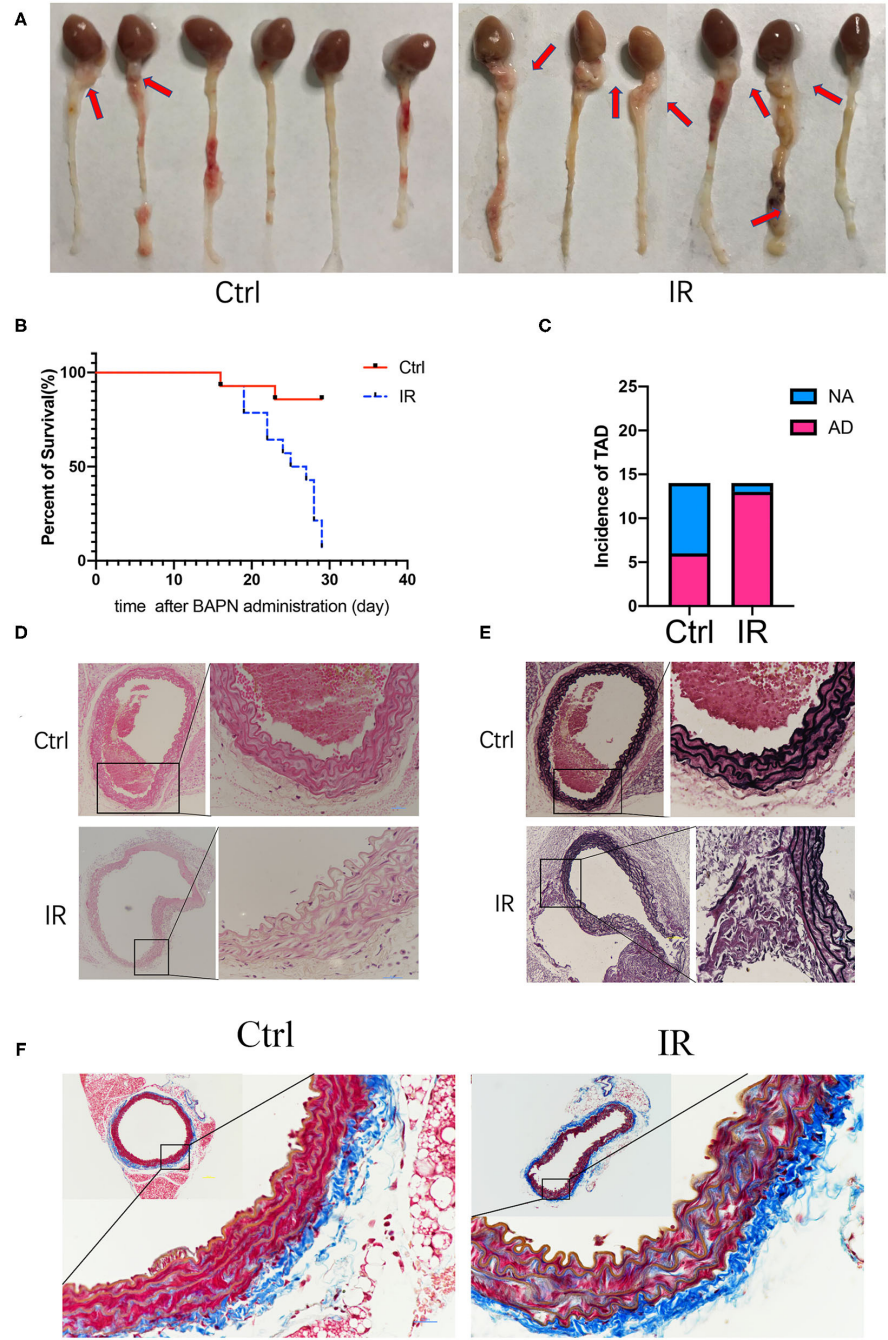
Beta-aminoproprionitrile (BAPN) induced the formation of AD in ApoE^−/−^ mice, reproducing major features of AD. **(A)** Representative images showing macroscopic features of isolated mouse aorta after different diets and BAPN treatment for 4 weeks; red arrow indicates the location of AD. **(B)** Kaplan-Meier survival curves. **(C)** The incidence of AD in the two groups. **(D)** Representative hematoxylin and eosin (H&E) staining showing the number of VSMCs was increased and the arrangement was disordered in the IR group. **(E)** Representative Verhöeff's staining shows that the elastic fiber layer becomes thinner and fibers smaller and broken in the IR group. **(F)** Masson staining showed that the collagen fibers increased in the IR group. Scale bars = 100 and 50 μm.

All mice had access to drinking water containing 0.25% BAPN, and the time of the occurrence of AD was recorded. The results showed that the IR group of mice had AD in 13 of 14, and 8 of them died from AD before using Ang-II; 6 of 14 of the mice in the normal diet group were diagnosed as AD and 2 of them died from AD before implanting the Ang-II pump (Figures 4A–C). The mice aorta were embedded in paraffin after fixation with 4% paraformaldehyde and dehydration. The sections were stained with H&E, elastic fiber, and collagenous fiber for observation under the microscope. Compared with the control group of mice, the number of smooth muscle cells increased in the IR group, and the arrangement was disordered and the basement membranes thinner. The extracellular matrix was increased, the elastic fiber layer was thinner with smaller, broken fibers, and there were more collagen fibers (Figures 4D–F).

#### Phenotype Switch of AVSMCs and MMPs Is Upregulated in the IR Group

**Figure 5:**
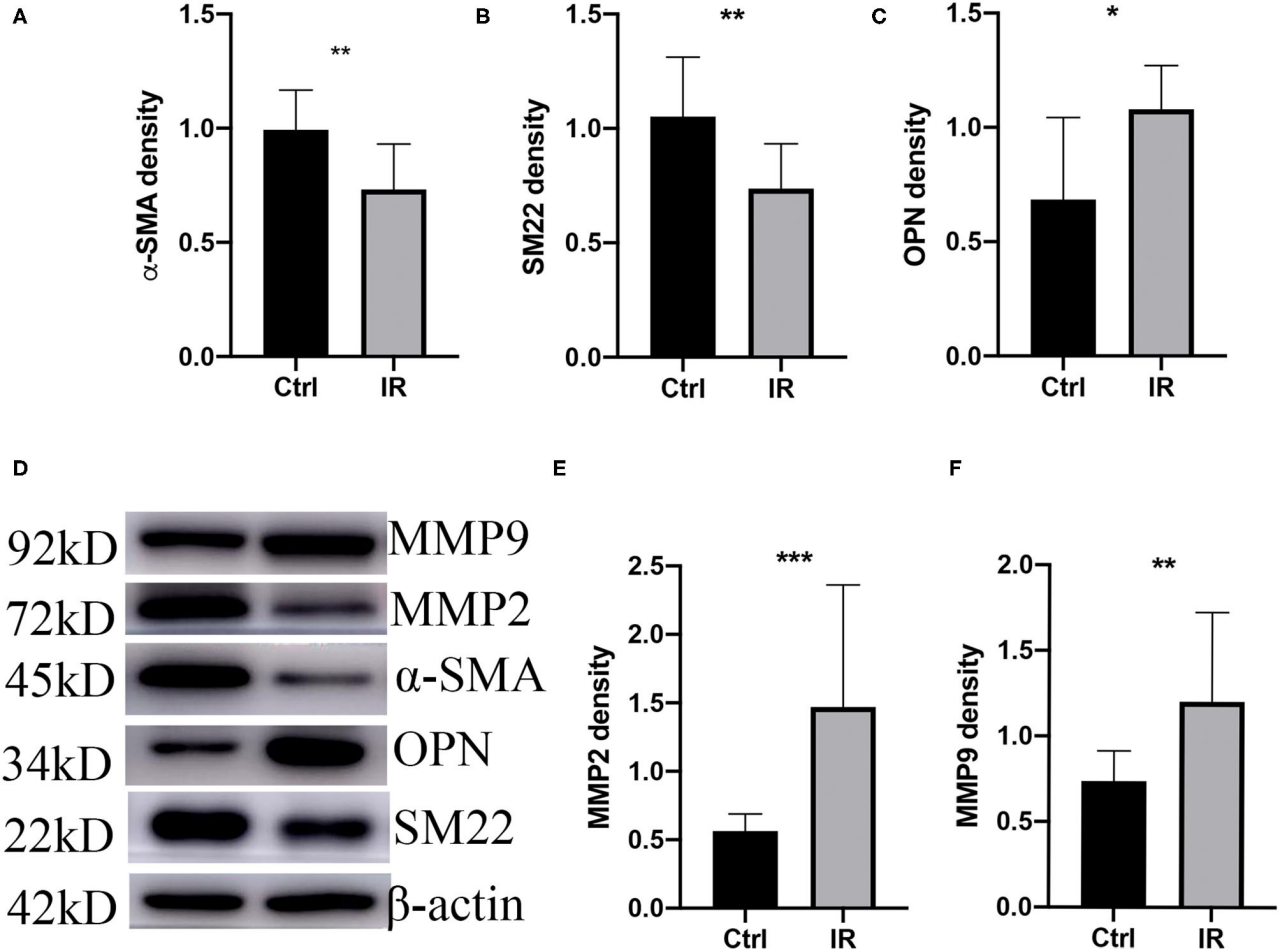
Phenotype switch of vascular smooth muscle cells (VSMCs) and upregulation of MMPs in mice. **(A)** α-SMA. **(B)** SM22. **(C)** OPN. **(D)** Westen blot (WB) assay of MMP9, MMP2, α-SMA, OPN, and SM22. **(E)** MMP2 and **(F)** MMP9 western blot normalized intensity relative to β-actin staining. The *p*-value of fine representative bands between two group was **P < 0.01, **P < 0.01, *P < 0.05, ***P < 0.001, and **P < 0.01, respectively.

The phenotypic switch of aortic vascular smooth muscle cells (AVSMCs) is a characteristic of AD; MMP2 and MMP9 are the most common metalloproteinases that promote AD. We extracted tissue proteins from fresh mouse aortic tissue and used western blot to quantity the protein expression of SM22, α-SMA, OPN, MMP2, and MMP9. The results showed that the protein expression of OPN, MMP2, and MMP9 (*P* < 0.05, *P* < 0.01, *P* < 0.001) was higher in the IR group, and that of SM22 and α-SMA (*P* < 0.01, *P* < 0.01) was lower (Figures 5A–F).

### Cell Experiments

#### Insulin Can Downregulate Glucose Consumption of HA-VSMCs and Lead to IR

**Figure 6:**
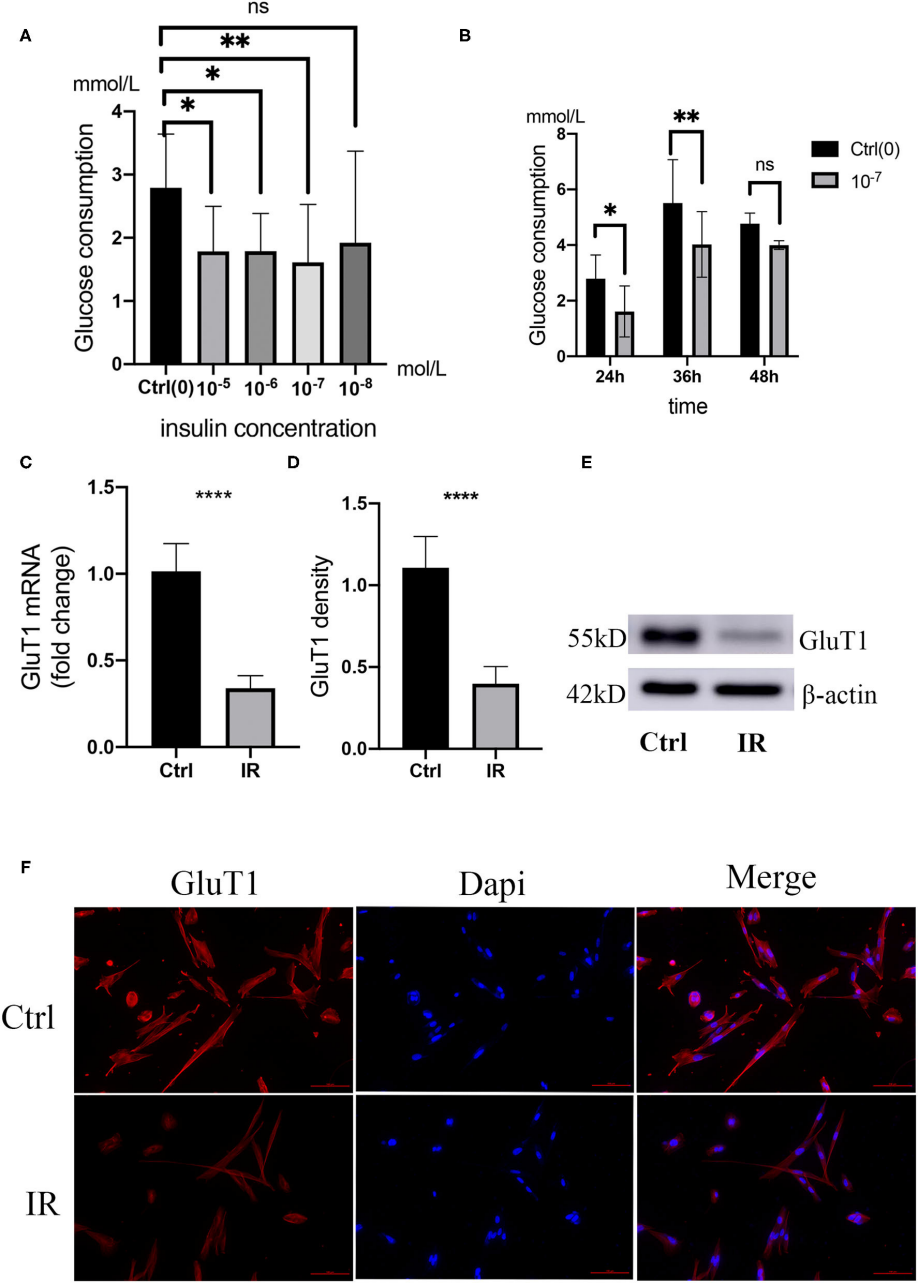
Establishment and verification of IR model in human aortic VSMCs (HA-VSMCs). **(A)** Glucose consumption in HA-VSMCs is downregulated with co-cultivation with different concentrations of insulin. *P < 0.05, **P < 0.01, NS, Not Statistically Significant. **(B)** Glucose consumption in HA-VSMCs is downregulated by co-cultivation with 10^−7^mol/L insulin at different times. **(C)** quantitative PCR (qPCR) analysis of GluT1 mRNA levels, ****P < 0.0001. **(D,E)** Western blots for GluT1 and β-actin stain considering Ctrl and IR cells, normalized intensity relative to β-actin staining. The *P*-value of three representative bands between two groups was **P < 0.01. **(F)** Immunofluorescence staining of GluT1 (red) in SMCs from the HA-VSMCs of the control and IR groups. DAPI staining was used to visualize cell nuclei. Scale bar = 100 μm.

Adding different concentrations of insulin to the culture medium can reduce the glucose consumption of HA-VSMCs, the glucose consumption of HA-VSMCs is downregulated with different concentrations of insulin in the culture medium and the 10^−7^ mol/L is the most effective suppression, which is downregulated with 10^−7^ mol/L insulin in the culture medium in a different time and the 36 h is the most effective suppression time (Figures 6A,B). To determine whether insulin can cause HA-VSMCs IR, we detected the mRNA expression of Glut1 in HA-VSMCs by qPCR, found that the mRNA expression of GluT1 in co-cultured with insulin was reduced (*P* < 0.0001) (Figure 6C). Then, we performed a western blot to verify the protein expression of Glut1 protein compared to the control group. We found that it was reduced in the IR group (*P* < 0.001) (Figures 6D,E) and found the same trend with immunofluorescence (Figure 6F).

#### IR Leads to a Phenotypic Switch of HA-VSMCs

**Figure 7:**
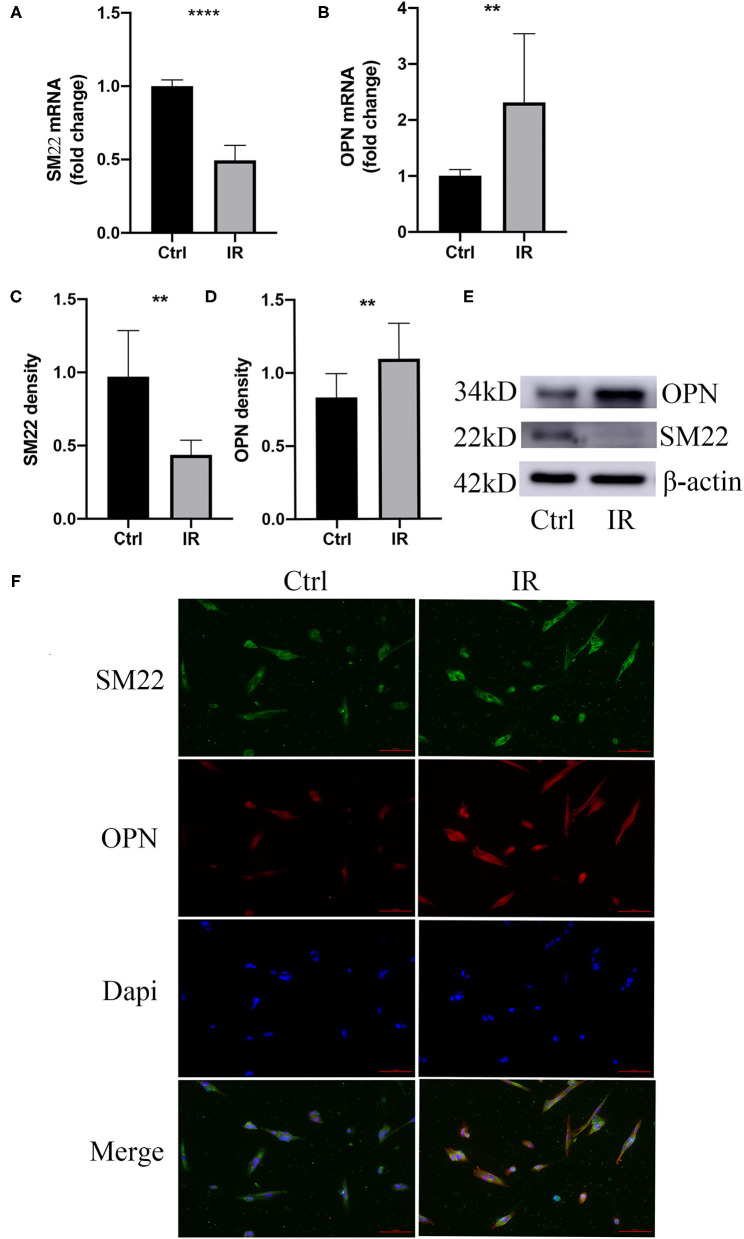
Phenotype switching of VSMCs in the cell model. **(A,B)** qPCR analysis of SM22 and OPN messenger RNA (mRNA) levels, **P < 0.01, ****P < 0.0001. **(C–E)** Western blots for SM22, OPN, and β-actin stain considering Ctrl and IR cells, this normalized intensity relative to β-actin staining, The *P*-value of three representative bands between the two groups was **P < 0.01 and ****P < 0.0001, respectively. **(F)** Immunofluorescence staining of SM22 (green) and OPN (red) in SMCs from the HA-VSMCs of the control and IR groups. DAPI staining was used to visualize cell nuclei. Scale bar = 100 μm.

To clarify that IR causes HA-VSMCs phenotype switching, we choose specific SM22 and OPN as indicators, using qPCR to detect mRNA expression and western blot to detect protein expression. The results showed that both mRNA and protein expression of SM22 in the IR HA-VSMCs decreased (*P* < 0.0001, *P* < 0.01) and those of OPN were upregulated (*P* < 0.01, *P* < 0.01) (Figures 7A–E). Results were further confirmed by immunofluorescence (Figure 7F), which indicated that IR can induce phenotypic switching of HA-VSMCs from the contractile to the synthetic phenotype.

## Discussion

Our study found that IR mainly attenuates the uptake of glucose by diminishing the expression of GluT1 membrane protein, induces the phenotypic switching of VSMCs from the contractile to the synthetic phenotype, enhances the protein expression of MMP2 and MMP9, and contributes to the occurrence of AD. Combined with the results of clinical analysis, verification of the protein and mRNA levels in human thoracic aorta, animal model, and cell experiments, we believe that IR is a high-risk factor for AD. This provides a basis for in-depth research on the specific mechanism of IR that promotes AD and a new approach for prevention and treatment.

Insulin resistance reduces the sensitivity of target cells to physiological levels of insulin concentration, and higher concentrations of insulin are then needed to maintain the balance between the supply and demand of nutrients (22, 23) leading to hyperglycemia and dyslipidemia (24, 25) and disturbed energy metabolism as a result of the reduced expression and function of the membrane protein GluTs. There are many methods to assess insulin sensitivity, including direct methods such as the hyper-insulinemic euglycemic clamp (HEC) (26), insulin suppression test (IST) (27), tracer detection (28), and indirect detection methods such as homeostasis model assessment (HOMA-IR) and beta-cell function index (HOMA-beta) (29), quantitative insulin sensitivity check index (QUICKI), and Bennett insulin sensitivity index (BISI) (30) among others. These methods are complicated or time-consuming, However, the occurrence and development of AAD are likely to be fatal within a short period of time, so rescue treatment is the best and most effective way to save lives. Therefore, the above methods are not suitable for assessing insulin sensitivity in patients with ADD. HbA1c is a relatively ideal indirect evaluation index, with low sensitivity and high specificity (31–33) that is minimally affected by the time of food intake and stress. So, in our study, we chose HbA1c as an indicator for evaluating insulin sensitivity. Studies have found that C-peptide, SIC, and HOMA-IR are significantly upregulated in patients with larger aneurysm diameters (20) and that elevated fructosamine and HbA1c levels are high-risk factors for AD (21). One clinical study used HOMA-IR as an indicator of insulin sensitivity, and the results showed that the elasticity of the ascending aorta and its overall properties in IR patients changed with time, with increased stiffness and decreased elastic expansion; this deterioration in those with mild IR was more obvious in a short period of time (34).

Our study found that the abnormal level of HbA1c in patients with ATAD was higher, and combined with the sensitivity and specificity of HbA1c in the diagnosis of AD, we believe that AD is a high-risk factor for ATAD. Human vascular smooth muscle cells transport glucose into the cell for metabolism, which mainly relies on the membrane protein GluT1 (35, 36). IR may lead to a decrease in GluT1 expression. To further verify this clinical phenomenon, we used q-PCR, western blot, and immunofluorescence to analyze the mRNA and protein expression of GluT1 in the VSMCs of patients with ATAD, which were significantly reduced, indicating that IR contributes to the decreased expression of GluT1 and the occurrence of ATAD.

We changed the diet of ApoE^−/−^ mice, monitored the FGC and SIC, assessed the insulin sensitivity of mice with HOMA-IR (37), and analyzed the protein expression of GluT1 in VSMC by western blot and immunofluorescence. We found higher HOMA-IR and lower protein expression of Glut1 in the high-fat diet group of mice, indicating that the animal model of IR was successfully established. We fed the mice 0.25% BAPN to induce AD (38). The results showed that the incidence of AD was significantly higher in the IR group. H&E, Masson, and elastin fiber staining were consistent with the pathology of the mouse model of AD investigated by others (39, 40). With protein analysis of aortic tissue in the two groups, we found that the expression of SM22 in VSMCs in the IR group was decreased and expression of OPN, MMP2, and MMP9 were increased, which showed that IR can promote the occurrence of AD and is a high-risk factor for AD.

We added different concentrations of insulin to the medium to establish insulin-resistant HA-VSMCs and determine the best insulin concentration and best action time for cellular IR (41, 42). We then verified the mRNA and protein expression of GluT1 by q-PCR and western blot, respectively. We found that the expression of SM22 was lower and OPN higher in insulin-resistant HA-VSMCs, which indicates IR caused the phenotypic switching of SMC from the contractile to the synthetic phenotype.

At present, most clinical studies have shown that diabetes has a protective effect against AD (43–45) and is negatively correlated with the enlargement and the rupture of the aneurysm (18, 46). Other studies showed that diabetes leads to hypertension and increases the frequency of AD (3), resulting in a higher frequency of hospitalization (47). Although a variety of oral hypoglycemic drugs that enhance insulin sensitivity have protective effects in reducing AD, insulin used to treat diabetes promoted the occurrence and development of AD (48). Through the analysis of clinical data, combined with the proportion of ATAD patients with abnormal HbA1c, it can be inferred that IR is a high-risk factor for ATAD. To clarify the relationship between IR and AD, it is necessary to carry out a related prospective clinical trial. We found that IR results in the phenotypic switch of VSMC, but the specific mechanism of action is still not clear, and further in-depth research is needed. This may provide a basis for reducing the occurrence and development of AD by actively treating IR, which may become a potential target for reducing the occurrence and treatment of AD and providing a new approach for its prevention and treatment.

## Conclusion

The results of this study show that IR induced phenotypic switching of SMCs from the contractile to the synthetic phenotype, which promotes the occurrence of AD. The study provides a basis for further research on the specific mechanism and a new approach for the active treatment of IR to reduce the occurrence of AD.

## Data Availability Statement

The datasets presented in this study can be found in online repositories. The names of the repository/repositories and accession numbers can be found in the article/Supplementary Material.

## Ethics Statement

The studies involving human participants were reviewed and approved by the Ethics Committee at the Fujian Medical University Union Hospital (No. 2021QH026). Written informed consent for participation was not required for this study in accordance with the national legislation and the institutional requirements. The animal study was reviewed and approved by the Laboratory Animal Care and Use Committee of School of Fujian Medical University (No. 2020-0089).

## Author Contributions

LC had full access to all the data in the study and takes responsibility for the integrity of the data and the accuracy of the data analysis. HZ was responsible for the study concept and design, acquisition of data, and data analysis and interpretation. HZ and CW conducted the experiments, data analysis, and statistical analysis and drafted the manuscript. TC and JY provided technical support and editing. JH, YZ, and XW were responsible for the investigation, tissues samples, and clinical data collection. ZQ provided supervision. LZ and YL provided administrative, technical, or material support. All authors contributed to the article and approved the submitted version.

## Funding

This study was supported by the National Natural Science Foundation of China (U2005202), the Natural Science Foundation of Fujian Province (2020J01998 and 2020J02056), and the Fujian Provincial Health Technology Project (2019-ZQN-50).

## Conflict of Interest

The authors declare that the research was conducted in the absence of any commercial or financial relationships that could be construed as a potential conflict of interest.

## Publisher's Note

All claims expressed in this article are solely those of the authors and do not necessarily represent those of their affiliated organizations, or those of the publisher, the editors and the reviewers. Any product that may be evaluated in this article, or claim that may be made by its manufacturer, is not guaranteed or endorsed by the publisher.
